# Absence of Oligoclonal Bands in Multiple Sclerosis: A Call for Differential Diagnosis

**DOI:** 10.3390/jcm12144656

**Published:** 2023-07-13

**Authors:** Evangelos Katsarogiannis, Anne-Marie Landtblom, Anna Kristoffersson, Johan Wikström, Robert Semnic, Shala G. Berntsson

**Affiliations:** 1 Department of Medical Sciences, Neurology, Uppsala University, SE-751 85 Uppsala, Sweden; evangelos.katsarogiannis@neuro.uu.se (E.K.); anne-marie.landtblom@neuro.uu.se (A.-M.L.); anna.kristoffersson@neuro.uu.se (A.K.); 2 Department of Surgical Sciences, Neuroradiology, Uppsala University, SE-751 85 Uppsala, Sweden; johan.wikstrom@radiol.uu.se (J.W.); rsemnic@gmail.com (R.S.)

**Keywords:** multiple sclerosis, oligoclonal bands, differential diagnosis

## Abstract

Background: Immunoglobulin gamma (IgG) oligoclonal bands (OCB) in the cerebrospinal fluid (CSF) are absent in a small group of multiple sclerosis (MS) patients. According to previous research, OCB-negative MS patients differ genetically but not clinically from OCB-positive MS patients. However, whether OCB-negative MS is a unique immunological and clinical entity remains unclear. The absence of OCB poses a significant challenge in diagnosing MS. (1) Objective: The objective of this study was twofold: (1) to determine the prevalence of OCB-negative MS patients in the Uppsala region, and (2) to assess the frequency of misdiagnosis in this patient group. (2) Methods: We conducted a retrospective study using data from the Swedish MS registry (SMSreg) covering 83% of prevalent MS cases up to 20 June 2020 to identify all MS patients in the Uppsala region. Subsequently, we collected relevant information from the medical records of all OCB-negative MS cases, including age of onset, gender, presenting symptoms, MRI features, phenotype, Expanded Disability Status Scale (EDSS) scores, and disease-modifying therapies (DMTs). (3) Results: Out of 759 MS patients identified, 69 had an OCB-negative MS diagnosis. Upon re-evaluation, 46 patients had a typical history and MRI findings of MS, while 23 had unusual clinical and/or radiologic features. An alternative diagnosis was established for the latter group, confirming the incorrectness of the initial MS diagnosis. The average EDSS score was 2.0 points higher in the MS group than in the non-MS group (*p* = 0.001). The overall misdiagnosis rate in the cohort was 33%, with 22% of misdiagnosed patients having received DMTs. (4) Conclusions: Our results confirm that the absence of OCB in the CSF should raise suspicion of possible misdiagnosis in MS patients and prompt a diagnostic reassessment.

## 1. Introduction

The intrathecal synthesis of oligoclonal IgG bands (OCB) is the most consistent immunological biomarker for MS, and it is present in the cerebrospinal fluid (CSF) in 90–95% of patients with relapsing-remitting MS (RRMS) [1,2]. However, it is well known that OCB status and clinical characteristics differ between Western and Eastern cases of MS and that Western MS has a higher rate of OCB-positivity [3], which is the focus of this study. OCB is less common in patients with clinically isolated syndrome (69%) but tends to appear in most patients with definite MS [1]. 

OCB-negative MS seems immunogenetically distinct from OCB-positive MS, with a dominance of HLA DR 4 instead of the expected HLA DR 15, as indicated in a large register-based study [4]. Nevertheless, both types share similar clinical characteristics, such as female predominance, age at onset, proportion of primary progressive cases, Magnetic Resonance Imaging (MRI) positivity rate, and disease severity [4]. No specific immunological features could be demonstrated in OCB-negative MS, as investigated by the cytokine mapping [5]. However, another study showed a lower tendency to develop progressive disease in OCB-negative MS [6]. Furthermore, specific MRI features have been proposed for OCB-negative MS, but the results have to be confirmed [7]. The scientific and clinical background of OCB-negative MS remains to be fully explained.

The absence of OCBs unique to the cerebrospinal fluid (CSF) does not exclude MS but should be considered a warning for potential misdiagnosis [8]. Conversely, the presence of OCBs, on the other hand, is no guarantee for an MS diagnosis as they can be present in CNS manifestations of other disorders, such as systemic lupus erythematosus, neurosyphilis, neuroborreliosis, HIV infection, paraneoplastic disorders, and neurosarcoidosis [9]. OCBs are also present in a small proportion of healthy individuals, including the healthy relatives of MS patients [10]. The most common differential diagnoses of MS include other neuroinflammatory white matter diseases, metabolic disorders, vasculopathy, and leukodystrophies [3]. 

The recent revision of McDonald’s criteria for diagnosing MS highlights the importance of MRI for MS diagnosis by emphasizing its sensitivity [11]. The availability of disease-modifying treatments (DMTs) has led to a call for early diagnosis, as there is evidence that early treatment can reduce long-term disability [12]. The highly potent DMTs require a robust diagnostic procedure due to potentially severe side effects and high treatment costs. Interestingly, the prominent role of MRI in the 2017 revision of the McDonald criteria may increase the risk of misdiagnosis, through the interpretation of non-specific or non-inflammatory demyelinating lesions, despite how radiological guidelines have evolved [13]. McDonald’s criteria are not intended to differentiate MS from other diseases. Therefore, other inflammatory CNS conditions should be excluded if a typical clinical presentation indicative of MS is not found [8,14]. 

Typical MRI features that support MS diagnosis, such as ovoid lesion shape, periventricular/juxtacortical localization, especially “Dawson’s fingers”, callosal atrophy, lesions at callososeptal interface, and an asymmetrical lesion distribution should also be applied to the diagnostic procedure, in addition to using the McDonald criteria [15,16]. Radiologic red flags include symmetrical pathology suggestive of leukodystrophy, infarcts/microbleeds suggestive of ischemic disease, and NMOSD-associated lesions [15]. 

The incidence of MS misdiagnosis varies significantly among studies ranging from 7% to 18% [17,18,19]. In one study, 79% of misdiagnosed patients received treatments initiated by a neurologist. Atypical clinical presentation, autoimmune diseases, and radiological red flags were associated with misdiagnosis [17].

The starting point for our study was the delayed diagnosis of two cases of leukodystrophy at Uppsala University Hospital, where both patients had previously been diagnosed with OCB-negative MS. Consequently, we conducted a retrospective cohort study of MS patients, focusing on all OCB-negative cases to further investigate the MS diagnosis and, where appropriate, search for differential diagnoses. The Swedish MS registry (SMSreg) retrieved data on all MS patients from the Uppsala region with negative OCB status. The study aimed to establish the frequency of OCB-negative MS patients in a local cohort, assess misdiagnosis of MS, and evaluate the effect of clinical and MRI findings on the predicting misdiagnosis. Hence, this study also serves as a retrospective quality control initiative to improve future diagnostic procedures.

## 2. Methods

This retrospective study includes all OCB-negative MS patients in the Uppsala region identified through a search in SMSreg until 20 June 2020, when the data were extracted [20]. Starting in 1996, the SMSreg constitutes a large national quality registry supported by governmental authorities. Currently, it covers more than 80% of prevalent MS patients at the national level. Retrospective registration was also conducted initially, including cases with onset before 1996, which affects the investigations performed and the diagnostic criteria applied. In 2019, the registration in Uppsala covered 83% of the presumed prevalent MS cases.

At the Department of Neurology at Uppsala University Hospital in Sweden, patient enrolment in the SMSreg is performed by MS specialists. 

Data collection in this study included the following parameters: demographic data, presenting symptoms, immunological data in terms of the presence or absence of oligoclonal band status and IgG index (normal reference ≤ 0.7) [21], age at disease onset, disease subtype (relapsing-remitting MS, secondary progressive MS, and primary progressive MS), radiological characteristics, treatment strategy (untreated, platform therapy, high-efficacy therapy), latest Expanded Disability Status Score (EDSS), and current relapse symptoms. CSF samples with two or more unique OCB bands than plasma is considered OCB positive. The OCB analysis was conducted using the electrophoresis procedure with an isoelectric focus on all study patients.

A prerequisite for a correct application of the McDonald criteria in MS is that they are used in syndromes typical for MS-related demyelination such as “unilateral optic neuritis, sensory deficits or weakness localizing to the spinal cord, cerebellar symptoms, or ophthalmoplegia with diplopia” [1]. The clinical diagnostic criteria for MS were standardized by Schumacher in 1965, and then CSF and evoked potentials were added by Poser in 1983. It was not until 2001 that the McDonald MRI criteria were added to the diagnostic procedure. Therefore, patients diagnosed before 2001 were not covered by the McDonald criteria but received their MS diagnosis based on clinical symptoms [22]. 

According to the 2017 McDonald MRI criteria, “a diagnosis of MS can be made when the criteria for dissemination in space (DIS) and dissemination in time (DIT) are met [11] in patients with clinical presentations typical for MS. Dissemination in space requires ≥1 T2 hyperintense lesions, symptomatic and/or asymptomatic, characteristic of multiple sclerosis in two or more of the following four locations: periventricular, cortical or juxtacortical, infratentorial, and the spinal cord”. “Dissemination in time can be determined by a new T2 hyperintense lesion compared to a previous MRI scan at baseline or the coexistence of a gadolinium-enhancing lesion and a non-enhancing T2 hyperintense lesion on an MRI scan”. “The detection of CSF-specific oligoclonal bands can replace the criterion of temporal spread”.

“The diagnosis of primary progressive MS requires ≥1 year of deterioration, which can be established either prospectively or retrospectively by two of the following”: 

“≥1 T2-hyperintense lesion characteristic of multiple sclerosis in one or more of the following regions: periventricular, cortical, or infratentorial”; “≥2 T2-hyperintense lesions in the spinal cord”; “Presence of CSF-specific oligoclonal bands”.

EDSS is a clinical scale used to grade MS-related disability. EDSS score is a composite of the disability rate of the different neurological functional systems affected by MS. Despite its limitations, EDSS is the most common outcome measure in MS clinical studies [23]. The clinical records of the patients were reviewed by three MS specialists (E.K, S.G.B, A-M. L), and previous MRI scans of the brain and spinal cord were evaluated by two experienced neuroradiologists.

### Statistical Analysis

Continuous variables were reported as means with standard deviations (SD). Dichotomous variables were analyzed using frequency distribution and presented as proportions. Comparisons between the MS and non-MS groups were assessed using the *t*-test. Nominal two-sided *p* < 0.05 was considered statistically significant. The statistical analysis was performed using IBM SPSS (version 28).

## 3. Results

A total of 759 patients from the Uppsala region were identified in the SMSreg from the start of the registry in 1996 until June 2020, of which 69 patients had an OCB-negative status (9%). After careful evaluation, the frequency of OCB-negative MS decreased to 6%. A lumbar puncture was performed once for the entire cohort (*n* = 69), with only five patients undergoing the procedure twice (*n* = 5).

Re-evaluation of the MS diagnosis based on clinical and radiologic findings resulted in two subgroups: an MS group consisting of 46 patients (67%) who fulfilled the clinical and radiologic McDonald criteria and a non-MS group consisting of 23 patients (33%) for whom a definitive MS diagnosis was questioned. The re-evaluation of some patients diagnosed several decades ago showed no updated MRI studies, but since the clinical course was consistent with MS, the diagnosis was left unchanged (*n* = 3). Some of the older cases had not been investigated with modern blood biomarkers such as aquaporin-4 and MOG antibodies since these were not available at that time. We considered this when scrutinizing the diagnoses.

The demographic data and clinical parameters of the whole sample are presented in Table 1. The mean age at MS onset was four years lower in the MS group compared to the non-MS group (35.9 vs. 39.8 years), but the difference was not statistically significant (*p* = 0.17). The gender distribution was similar in the two groups. Sensory deficits were the most common presenting symptom in both groups (32% vs. 48%), followed by weakness (30% vs. 17%) and optic neuritis (19% vs. 16%). The mean IgG index was 0.65 for the MS group and 0.53 for the non-MS group (*p* = 0.12)

The MS phenotype was mainly RRMS in both groups (45% and 65%), and the prevalence of primary progressive multiple MS (PPMS) and secondary progressive MS (SPMS) did not differ significantly between the two groups. The mean EDSS score was considerably higher in the MS group than in the non-MS group (*p* = 0.001). In the MS group, 58% achieved an EDSS score of 3, and 45% had an EDSS score of 6, while in the non-MS group, 30% achieved an EDSS score of 3, but only 9% had an EDSS score of 6 or more. 

Regarding the presence of inflammatory/autoimmune diseases in the sample, we found two cases with type 1 diabetes, one with sarcoidosis and one with ankylosing spondylitis. Many patients did not receive DMTs in the MS group (52%) and in the non-MS group (78%). Platform DMTs (interferons, glatiramer acetate, dimethyl fumarate, teriflunomide) had previously been used with 32% of the MS group and 17% of the non-MS group. Notably, only one patient belonging to the non-MS group had ongoing or previous treatment with more potent drugs (natalizumab, alemtuzumab, cladribine, fingolimod, rituximab, autologous stem cell transplantation) compared to 7 patients (15%) in the MS group. The demographic and clinical parameters for the non-MS group are illustrated in Table 2. Several patients who showed neither clinical relapses nor signs of progressive disease were labeled “benign MS” in the clinical files. All patients in the non-MS group had an alternative or concurrent diagnosis according to the ICD-10 system, representing a 33% misdiagnosis rate in the cohort (Table 2).

Table 3 summarizes the radiological characteristics of the 23 patients in the non-MS group. White matter lesions (WML) with morphology inconsistent with MS or suggestive of small vessel disease were a common finding in this group. Six patients had a normal brain MRI. 

## 4. Discussion

The frequency of OCB-negative MS patients in the Uppsala cohort identified from SMSreg was initially 9%. However, after re-evaluation, the frequency decreased to 6%, in line with previous studies showing a frequency between 5–10% [1,2,4]. 

Furthermore, we found that 33% of patients received a diagnosis other than MS after re-evaluating clinical and radiological data. This is in line with a large study from 22 MS centers, showing that the absence of OCBs, atypical lesions on MRI, lack of dissemination in space, and normal visual evoked potentials was equally predictive of a diagnosis other than MS [24]. In the revised 2017 McDonald criteria, OCB positivity was considered to meet the criterion for dissemination in time, and the role of OCBs as an independent predictor of a second clinical attack was also emphasized [21,25]. 

It is important to note that our study investigated a very long-term cohort from 1996 to 2020, meaning that the first MS cases were diagnosed using older diagnostic criteria, i.e., Schumacher and Poser, before MRI became a standard procedure [19]. At that time, before the development of the MacDonald criteria, clinical evidence was most important. This may have affected the quality of the diagnostic procedure in our cohort. 

### 4.1. The McDonald Criteria and Radiology

The McDonald criteria “were not developed to distinguish MS from other conditions”, but to confirm an early diagnosis of MS in patients presenting with typical demyelinating syndromes [8,14]. Applying the criteria to patients presenting with atypical syndromes (presentations other than optic neuritis, brainstem/cerebellar syndromes, or myelitis) lowers the accuracy of the MS diagnosis [26]. In our cohort, this was initially highlighted by the two patients with leukodystrophy, who had been misdiagnosed as OCB-negative MS. Adult-onset leukodystrophies pose a clinical and radiological diagnostic challenge due to the wide phenotypic spectrum, age of onset, and patterns of white matter lesions [27]. 

Differentiating these inherited white matter disorders from MS requires the recognition of specific hypomyelination patterns on MRI [27,28]. A secondary review of the MRI data played an essential role in our study. The fact that several of our patients with atypical MRI had received an incorrect MS diagnosis and were entered into the SMSreg might be seen as an inclusion bias, but this should be considered from the perspective of real-world data and clinical practice. We sometimes encounter medical cases that are difficult to classify, which can lead to ambiguity in a register if the criteria are not followed. Importantly, McDonald’s criteria have a modest specificity and, therefore, a low ability to distinguish MS from other degenerative or autoimmune white matter diseases [13]. The 2017 criteria revision enabled dissemination in space with fewer lesions compared to previous versions. Repeated MRI scans and CSF studies to confirm the presence of CSF-restricted OCB, or waiting for a new relapse typical of MS, are necessary to confirm the diagnosis of MS in patients presenting with atypical syndromes or red flags [29]. 

### 4.2. OCB as Biomarkers

OCBs in MS are associated with both CNS inflammation and disability. However, whether OCBs play a role in disease pathogenesis remains to be fully understood [30,31]. 

The important role of B cells in the immunopathology of MS is supported by studies showing antibody-mediated oligodendrocyte and axonal injury in MS [21]. The presence of OCBs is another marker of the importance of B cells in MS, as they are produced by plasma cells [32]. The prognostic role of OCBs is implicated in reports on the presence of B-cell follicles containing plasma cells in patients with severe disease progression [33]. However, the precise role of OCBs in MS-related inflammation is not well understood, and OCB negativity does not exclude an ongoing inflammatory process. An elevated IgG index (>0.7 at disease onset) is associated with OCB positivity and is prognostic of the disease activity [34]. The recent lymphoid chemokine CXCL 13 biomarker is a reliable predictor of activity in MS patients [35]. A similar prognostic value was shown for CSF kappa-free light chains (KFLC), indicating an intrathecal IgG synthesis [36]. Despite their diagnostic and prognostic value, these biomarkers are not currently incorporated into clinical practice or included in the 2017 McDonald criteria. Consequently, they were not specifically evaluated in our retrospective study.

### 4.3. PPMS Diagnosis—A Challenge

Six of the 23 patients in the non-MS group received a PPMS diagnosis. This is a challenging diagnosis that requires a qualified differential diagnosis. The prevalence of OCBs in PPMS varies in different studies. For example, one study found that 21% of PPMS patients were OCB negative in the cerebrospinal fluid (CSF), while a recent study using the SMSreg data showed a comparable OCB status in PPMS and relapsing-remitting MS (RRMS) [4,37]. The 2017 revised McDonald criteria includes patients with PPMS [14], and, importantly, the presence of CSF-specific OCB remains an additional criterion for diagnosing PPMS. In Sweden, where the CSF examination is routinely performed, meeting this criterion is relatively straightforward. However, in many other countries where lumbar puncture is not routinely performed, diagnosing PPMS can be more challenging due to the absence of CSF-specific OCB testing.

### 4.4. Benign MS

Most patients misdiagnosed with MS in our sample had a relatively mild and prolonged disease course and were followed up by the same physician over many years. This may indicate the challenge of forming an alternative diagnosis in patients with the same level of neurological dysfunction over a long period. On the other hand, some patients in the non-MS group were referred to two or more specialists after being diagnosed with MS. This is consistent with previous data suggesting that neurologists are reluctant to re-evaluate an existing MS diagnosis [38]. “Benign MS” is often used to describe a disease course without significant disability accumulation.

An EDSS score of 3 or less after ten years of disease is the most common definition of benign MS [39]. Although some disability progression might be expected, the longer the patient remains benign, the lower the final EDSS score at an advanced age. In our study, the non-MS group displayed a low mean EDSS score of 2.5; only 7 of the 23 patients (30%) reached an EDSS score of 3 or higher. Benign MS was a common diagnosis in this subgroup, leading to relatively little follow-up and no further re-evaluation of the disease. 

### 4.5. Criteria for Treatment

The development of modern, highly effective DMTs has increased the move toward the early detection of MS, including using the McDonald criteria. 

In our study, 22% of the misdiagnosed patients had received immunomodulating drugs. However, they were less likely to be treated with DMTs than OCB-negative MS patients, underlining the importance of individualized treatment decisions and goals. It is essential to consider the potential risks of treatment in patients who do not have MS, as side effects can be severe. For instance, rituximab-treated patients have been found to have an increased risk of severe COVID-19 [40]. The diagnostic criteria for MS were modified when the patients in our study were included in the MS registry, thus accounting for inclusion bias.

## 5. Conclusions

In our analysis of the misdiagnosed cases, we did not find any specific characteristics that distinguished the two groups, except for a lower Expanded Disability Status Scale (EDSS) score and a lower IgG index. Some of these cases were labeled as “benign MS”, which may have contributed to the lack of re-examination.

In addition, one-fifth of the patients in our cohort who were misdiagnosed had received immunomodulatory drugs, which increases the risk of side effects. 

Furthermore, our identification of two cases of hereditary leukodystrophy highlights the potential risk of missing genetic diseases, leading to delays in genetic screening and counseling for affected individuals and their relatives.

In conclusion, this real-world cohort study highlights the importance of OCB negativity of CSF in the misdiagnosis of MS. A high suspicion of an alternative diagnosis other than MS should be retained for OCB-negative patients. Importantly, the implications of the McDonald criteria in patients with atypical clinical presentations may lead to misdiagnosis. We recommend re-evaluating patients with an old OCB-negative MS diagnosis over time to exclude the possibility of a non-MS diagnosis.

## Figures and Tables

**Table 1:** Demographic data and clinical parameters of the study population of OCB-negative patients (*n* = 69).

Variables	MS	Non-MS	*p*-Value
Patients (*n* = 69)	46 (66%)	23 (34%)	
Age at onset (year), mean (SD)	35.9 (9.8)	39.8 (11.3)	0.55
Female/male	32/14	18/5	0.11
Presenting symptoms			
Sensory (%)	15 (32)	11 (48)	
Myelitis (%)	1 (2)	1 (4)	
Brainstem (%)	4 (9)	1 (4)	
Optic neuritis (%)	9 (19)	4 (17)	
Ataxia (%)	2 (4)	2 (8)	
Motor (%)	14 (30)	4 (17)	
Phenotype	MS	Non-MS	0.84
RR (%)	21 (45)	15 (65)	
PP (%)	14 (30)	6 (26)	
SP (%)	11 (24)	2 (9)	
EDSS, mean (SD)	4.5 (3.2)	2.5 (2.3)	0.001
Reached EDSS 3 (%)	27 (58)	7 (30)	
Reached EDSS 6 (%)	21 (45)	2 (9)	
CSF			
Age at CSF examination, mean (SD)	39.8 (9.9)	42.7 (11.6)	0.42
IgG Index, mean (SD)			
Ref (0.36−0.56) mg/dL	0.65 (0.3)	0.53 (0.16)	0.12
Treatment			
None (%)	24 (52)	18 (78)	
Platform DMT (%)	15 (32)	4 (17)	
More potent DMT (%)	7 (15)	1 (4)	

SD: standard deviation, CSF: cerebrospinal fluid, DMT: disease-modifying treatment, PP: primary progressive, RR: relapsing-remitting, SP: secondary progressive, EDSS: Expanded Disability Status Scale.

**Table 2:** Demographic and clinical data of patients in the non-MS group (*n* = 23).

Case	Age at Onset, y/Sex	MS-Subtype in SMSreg	Presenting Symptoms	EDSS	DMT	Time to Alternative Diagnosis	Revised Diagnosis
1	54/F	SPMS	Motor	5.5	No	24	Normal pressure hydrocephalus
2	66/F	RRMS	Myelitis	1.0	No	-	Non-inflammatory myelitis
3	33/F	RRMS	Dizziness	1.0	No		Vertigo NUD
4	43/M	PPMS	Brainstem	3.0	No	10	Arnold-Chiari malformation
5	48/F	PPMS	Motor	7.5	IFN	-	Gait difficulties
6	59/M	RRMS	Sensory	1.0	No	-	Cervical disc herniation
7	59/M	PPMS	Gait	5.5	No	5	Mild cognitive impairment
8	35/F	RRMS	Sensory	2.5	INF		Paresthesia
9	30/F	RRMS	Sensory	4.5	No	33	Leukodystrophy
10	30/F	RRMS	Sensory	0.0	No	-	Vertigo
11	35/F	PPMS	Sensory	2.0	No	21	Adrenoleuko-dystrophy
12	36/F	RRMS	ON	0.0	No	-	Optic neuritis
13	41/F	PPMS	Sensory, fatigue	1.5	No	-	Fatigue
14	26/F	RRMS	ON	2.5	IFN	-	Optic neuritis
15	30/F	RRMS	Sensory	0.0	No	-	Numbness of the limbs
16	22/F	RRMS	Sensory	0.0	No	-	Numbness in the face
17	28/M	RRMS	Motor	3.5	No	-	Hemiparesis NUD
18	40/F	RRMS	Sensory	0	No	-	Possible NMO
19	39/F	RRMS	Sensory	1.5	No	-	Myelitis
20	49/F	RRMS	Brainstem	5.0	NTZ	2	NMO
21	42/M	RRMS	ON	1.5	No	-	Optic neuritis
22	35/F	PPMS	Sensory	6.5	No	-	Hereditary spastic paraparesis
23	37/F	RRMS	ON	0	INF	-	Optic neuritis

Abbreviations: DMT: disease-modifying therapy; PPMS: primary progressive MS; RRMS: relapsing-remitting MS; SPMS: secondary progressive MS; EDSS: Expanded Disability Status Scale; IFN: Interferons; NTZ: Natalizumab; SMSreg: Swedish MS registry; NMO: neuromyelitis optica.

**Table 3:** The radiological characteristics of patients in the non-MS group (*n* = 23).

Case	Age at Onset (y)/Sex	MRI of the Brain	MRI of the Spinal Cord	Revised Diagnosis
1	54/F	Supra- and infratentorial WM lesions (WML) suggest small vessel disease. Compressed parasagittal sulci, dilated ventricles, fissure sylvii, and convexity sulci suggestive of NPH	Normal	Normal pressure hydrocephalus
2	66/F	Left single frontal WML	8 mm C2- lesion	Non-inflammatory myelitis
3	33/F	Symmetrical periventricular WML suggestive of small vessel disease	Normal cervical MRI	Vertigo
n4	43/M	Five non-specific WML	Arnold-Chiari malformation	Arnold-Chiari malformation
5	48/F	Multiple, partially confluent signal intensity changes in WM suggest small vessel disease. Ischemic lesions in the basal ganglia.	Cervical and thoracic spinal cord normal	Gait difficulties
6	59/M	Symmetrical small WML. One large lesion in left temporal WM	Cervical disc herniation	Cervical disc herniation
7	59/M	Symmetrical periventricular WML suggestive of small vessel disease and non-specific WML. Cortical infarctions parietally and in the left cerebellar hemisphere	Normal cervical MRI	Mild cognitive impairment
8	35/F	Symmetrical periventricular WML suggestive of small vessel disease and non-specific WML	Normal cervical MRI	Paresthesia
9	30/F	Non-specific WML. Pons and cerebellar oedema	Cervical spine oedema	Leukodystrophy
10	30/F	Normal	Missing	vertigo
11	35/F	Non-specific WML. Corona radiata gliosis	Stripe-formed lesion in the posterior cervical medulla.	Adrenoleuko-dystrophy
12	36/F	7 non-specific supratentorial WML	Missing	Optic neuritis
13	41/F	Symmetrical confluent WML. Gliosis	Normal cervical MRI	Fatigue
14	26/F	Normal	Normal cervical MRI. Artefact at C3 level	Optic neuritis
15	30/F	Normal	Normal cervical and thoracical MRI	Numbness in the limbs
16	22/F	Normal	Normal cervical and thoracic MRI.	Numbness in the face
17	28/M	2 periventricular WML	Normal cervical and thoracical MRI	Hemiparesis NUD
18	40/F	Normal	C3, C4 lesions. GD+ C3 lesion	Possible NMO
19	39/F	Non-specific WML	C2, C4–5, C5–6 lesions. GD+ C2, C5-C6 lesion	Myelitis
20	49/F	GD+ medulla obongata lesion	GD+ C1–2 lesion	NMO
21	42/M	2–3 non-specific WML	Missing	Optic neuritis
22	35/F	Normal	Normal	Hereditary spastic paraparesis
23	37/F	10 non-specific WML	Normal cervical and thoracical MRI	Optic neuritis

## Data Availability

The data supporting this study’s findings are available from the corresponding author upon reasonable request.
